# Interactive evolutionary computation with minimum fitness evaluation requirement and offline algorithm design

**DOI:** 10.1186/s40064-016-1789-1

**Published:** 2016-02-27

**Authors:** Hisao Ishibuchi, Takahiko Sudo, Yusuke Nojima

**Affiliations:** Department of Computer Science and Intelligent Systems, Graduate School of Engineering, Osaka Prefecture University, 1-1 Gakuen-cho, Naka-ku, Sakai, Osaka 599-8531 Japan

**Keywords:** Interactive evolutionary computation, Interactive algorithms, Automatic algorithm design, Meta-level evolutionary algorithms

## Abstract

In interactive evolutionary computation (IEC), each solution is evaluated by a human user. Usually the total number of examined solutions is very small. In some applications such as hearing aid design and music composition, only a single solution can be evaluated at a time by a human user. Moreover, accurate and precise numerical evaluation is difficult. Based on these considerations, we formulated an IEC model with the minimum requirement for fitness evaluation ability of human users under the following assumptions: They can evaluate only a single solution at a time, they can memorize only a single previous solution they have just evaluated, their evaluation result on the current solution is whether it is better than the previous one or not, and the best solution among the evaluated ones should be identified after a pre-specified number of evaluations. In this paper, we first explain our IEC model in detail. Next we propose a ($\mu +1$)ES-style algorithm for our IEC model. Then we propose an offline meta-level approach to automated algorithm design for our IEC model. The main feature of our approach is the use of a different mechanism (e.g., mutation, crossover, random initialization) to generate each solution to be evaluated. Through computational experiments on test problems, our approach is compared with the ($\mu +1$)ES-style algorithm where a solution generation mechanism is pre-specified and fixed throughout the execution of the algorithm.

## Background

Interactive evolutionary computation (IEC) is a class of evolutionary algorithms, which are based on subjective fitness evaluation by a human user (Takagi 2001). IEC is a promising research area in the field of evolutionary computation (EC). In IEC, no explicit fitness function is assumed since each solution is subjectively evaluated by a human user. A number of successful applications of IEC have been reported in the literature (Arevalillo-Herráez et al. 2011; Cho 2002, 2004; Kim and Cho 2000; Lai and Chen 2011; Lameijer et al. 2006). In a typical scenario of IEC, a small number of solutions (e.g., a population of ten solutions) are shown to a human user. He/she is supposed to assign one of a pre-specified set of ranks (e.g., 1: very bad, 2: bad, 3: average, 4: good, 5: very good) to each solution in the population. In this scenario, it is implicitly assumed that a human user can evaluate multiple solutions at a time. It is also assumed that a human user can assign a different rank to each solution. However, it is not always easy to assign a different rank to each solution. A simpler fitness evaluation scheme is the choice of a pre-specified number of good solutions from a population (e.g., to choose three from a population of ten solutions). The simplest setting under this scheme is a pair-wise comparison where two solutions are compared with each other (i.e., a better solution is selected from the presented two solutions). In pair-wise comparison-based IEC models (Fukumoto et al. 2010; Takagi and Pallez 2009), it is implicitly assumed that two solutions can be evaluated simultaneously. Thus, the comparison of two solutions is usually counted as a single evaluation. However, in some application tasks of IEC such as hearing aid design (Takagi and Ohsaki 2007) and music composition (Fernandez and Vico 2013), human users can evaluate only a single solution at a time. Our focus in this paper is such a situation where a pair-wise comparison is counted as two evaluations.

In this paper, we assume the following simplest fitness evaluation scenario: a single solution is evaluated at a time, the current solution is compared with the previous one that has been just evaluated, and the evaluation result is whether the current solution is better than the previous one or not. Based on this scenario, we formulated an IEC model with the minimum requirement for the fitness evaluation ability of human users (Ishibuchi et al. 2012, 2014a, b). More specifically, our IEC model is based on the following assumptions:

(i) A human user can evaluate only a single solution at a time.
(ii) A human user can memorize only a single previous solution. After the evaluation of a current solution is completed, his/her memory is replaced with the newly evaluated one independent of its evaluation result.
(iii) A human user can evaluate the current solution in comparison with the previous solution in his/her memory. The evaluation result is whether the current solution is better than the previous one or not.
(iv) A human user can evaluate a pre-specified number of solutions in total.
  In addition to these assumptions, we further assume that the following requirement should be satisfied in order to identify a single final solution (Ishibuchi et al. 2012, 2014a, b):
(v) When a pre-specified number of evaluations is completed, the best solution among the evaluated ones should be identified.

One important issue in IEC is to decrease the burden of a human user in fitness evaluation (Sun et al. 2012). Our IEC model was formulated for this purpose by assuming the minimum requirement for human user’s fitness evaluation ability. As a result, the complexity of a human user’s response is minimized. That is, a human user in our IEC model is supposed to answer the following yes-or-no question after the evaluation of each solution: “Is the current solution better than the previous one?” The simplicity of a human user’s response may lead to the possibility of its automated recognition from his/her facial expression or brain wave activity in the future. This recognition task in our model is much simpler than the case of a five-rank evaluation scheme. It may be very difficult to automatically classify a human user’s reaction into one of the five ranks. The use of the simple fitness evaluation scheme in our IEC model will make the automated recognition task much easier. Our future goal is the implementation of an IEC model with an automated recognition system. However, in this paper, we focus on the design of evolutionary algorithms to efficiently search for a good solution using a simple fitness evaluation scheme: Whether the current solution is better than the previous one or not.

This paper is an extended version of our former conference papers (Ishibuchi et al. (2012, 2014a, b)). In Ishibuchi et al. (2012), we proposed the basic idea of our IEC model with the minimum requirement for human user’s fitness evaluation ability. We also implemented a simple evolutionary algorithm for our IEC model, which was based on the $(1+1)$ generation update mechanism of evolution strategy (ES). This algorithm was referred to as the $(1+1)$ ES-style algorithm. In Ishibuchi et al. (2014a), we generalized the $(1+1)$ES-style algorithm to a $(\mu +1)$ ES-style algorithm by proposing an archive maintenance mechanism, which was used to decrease the archive size from $\mu$ to 1 before the termination of the algorithm. Then we proposed an idea of automatically designing an evolutionary algorithm for our IEC model in Ishibuchi et al. (2014b). Our idea was to use an offline meta-level approach for the design of an IEC algorithm. An IEC algorithm was designed by specifying an operator (e.g., crossover, mutation, and random initialization) to generate each solution. In Ishibuchi et al. (2014b), an IEC algorithm with 200 evaluations was represented by an operator string of length 200. The *i*-th operator in each string was used to generate a solution for the *i*-th evaluation ($i = 1, 2,\ldots , 200$). Each string was evaluated by applying it to a test problem 100 times. In this paper, we examine the effect of the following factors on the performance of automatically designed algorithms through computational experiments on a number of test problems:

### The number of runs used for evaluating each string

Due to a stochastic nature of EC algorithms, usually a different solution is obtained from a different run of the same EC algorithm. Thus its performance evaluation needs multiple runs. This means that the fitness evaluation of a string in our offline meta-level approach needs multiple runs of the corresponding IEC algorithm. In this paper, we examine the relation between the number of runs for fitness evaluation and the performance of designed algorithms.

### The string length

In Ishibuchi et al. (2014b), an IEC algorithm with 200 evaluations was coded by an integer string of length 200 where each integer shows an operator for generating a single solution. If we use six candidate operators as in Ishibuchi et al. (2014b), the size of the search space (i.e., the total number of different strings) is $6^{200}$. Since the search space is large and the fitness evaluation has a stochastic nature, it is not likely that the optimal solution can be obtained. For the same reason, it is not easy to search for a good approximate solution, either. A simple idea for decreasing the size of the search space is the use of the same operator to generate a number of solutions. For example, if the same operator is used to generate 20 solutions, an IEC algorithm with 200 evaluations is coded by an integer string of length 10. The search space is decreased from $6^{200}$ to $6^{10}$. The first value of the string of length 10 is used to generate the first 20 solutions. In this paper, we examine the relation between the string length and the performance of designed algorithms.

### The number of possible operators

In Ishibuchi et al. (2014b), one of six candidate operators was selected to generate a single solution. Other specifications of candidate operators can be possible. For example, we can use a sequence of operators such as “crossover & mutation” and “mutation & mutation” as a single candidate operator to generate a new solution. In this manner, we can increase the number of candidate operators for generating a solution. It is also possible to decrease the number of candidate operators by removing a specific operator (e.g., crossover). In this paper, we examine the relation between the specification of candidate operators and the performance of designed algorithms.

In this paper [and in our former studies (Ishibuchi et al. 2012, 2014a, b)], we use a test problem instead of a human decision maker in computational experiments. No actual IEC experiments with human decision makers are included. Practical usefulness of our offline meta-level approach totally depends on the similarity between an actual IEC problem and a test problem used in our computational experiments. Our intention is not to insist any practical usefulness of our approach in real-world IEC applications, but to discuss the design of IEC algorithms under severely limited information about the fitness of each solution. We believe that the idea of using a different operator to generate each generation will give a new insight to the design of IEC algorithms and also to the design of EC algorithms in general.

This paper is organized as follows. In “Our IEC model” section, we explain our IEC model. In “[Sec Sec6]” section, we show how an archive maintenance mechanism in our former study (Ishibuchi et al. 2014b) was derived. Using the derived mechanism, we explain our ($\mu +1$)ES-style algorithm in its general form including the case of $\mu = 1$. Its performance is also examined in “[Sec Sec6]” section for different values of $\mu$. In “Meta-level approach to the design of IEC algorithms” section, we show an offline meta-level approach for automatically designing an IEC algorithm. The performance of designed algorithms under various settings of our offline meta-level approach is also evaluated in comparison with the ($\mu +1$)ES-style algorithm in “Meta-level approach to the design of IEC algorithms” section. This paper is concluded in “Conclusion” section.

## Our IEC model

The main feature of our IEC model is the necessity of solution re-evaluation for identifying the best solution among the evaluated ones. Some solutions may be re-evaluated several times. This is often the case in our everyday life. For example, we usually examine some pairs of glasses several times to compare them with each other before buying a single pair. It is very difficult for us to choose a single best solution after evaluating a number of solutions just once. Let us explain this feature using the following simple example with five solutions.

### *Example 1*

Ishibuchi et al. (2014b) Let us assume that we have five solutions: $\mathbf{x}^\mathrm{A}, \mathbf{x}^\mathrm{B}, \mathbf{x}^\mathrm{C}, \mathbf{x}^\mathrm{D}, \mathbf{x}^\mathrm{E}$. We also assume that $\mathbf{x}^\mathrm{C} \prec \mathbf{x}^\mathrm{B} \prec \mathbf{x}^\mathrm{A} \prec \mathbf{x}^\mathrm{E} \prec \mathbf{x}^\mathrm{D}$ holds where $\mathbf{x} \prec \mathbf{y}$ means that a solution $\mathbf{y}$ is preferred to a solution $\mathbf{x}$. Thus $\mathbf{x}^\mathrm{C}$ is the worst and $\mathbf{x}^\mathrm{D}$ is the best. Let us evaluate the five solutions $\mathbf{x}^\mathrm{A}, \mathbf{x}^\mathrm{B}, \mathbf{x}^\mathrm{C}, \mathbf{x}^\mathrm{D}$ and $\mathbf{x}^\mathrm{E}$ in this alphabetical order. First $\mathbf{x}^\mathrm{A}$ is shown to a human user. Next $\mathbf{x}^\mathrm{B}$ is evaluated in comparison with $\mathbf{x}^\mathrm{A}$. The evaluation result is “$\mathbf{x}^\mathrm{A}$ is better than $\mathbf{x}^\mathrm{B}$ (i.e., $\mathbf{x}^\mathrm{B} \prec \mathbf{x}^\mathrm{A}$)”. Then $\mathbf{x}^\mathrm{C}$ is evaluated as $\mathbf{x}^\mathrm{C} \prec \mathbf{x}^\mathrm{B}$. After the evaluation of the three solutions, we can say that $\mathbf{x}^\mathrm{A}$ is the best since $\mathbf{x}^\mathrm{C} \prec \mathbf{x}^\mathrm{B} \prec \mathbf{x}^\mathrm{A}$ holds from the evaluation results $\mathbf{x}^\mathrm{B} \prec \mathbf{x}^\mathrm{A}$ and $\mathbf{x}^\mathrm{C} \prec \mathbf{x}^\mathrm{B}$. Then $\mathbf{x}^\mathrm{D}$ is evaluated as $\mathbf{x}^\mathrm{C} \prec \mathbf{x}^\mathrm{D}$. After the evaluation of $\mathbf{x}^\mathrm{D}$, we cannot say which is the best between $\mathbf{x}^\mathrm{A}$ and $\mathbf{x}^\mathrm{D}$ (since the available information is $\mathbf{x}^\mathrm{C} \prec \mathbf{x}^\mathrm{B} \prec \mathbf{x}^\mathrm{A}$ and $\mathbf{x}^\mathrm{C} \prec \mathbf{x}^\mathrm{D}$). Finally $\mathbf{x}^\mathrm{E}$ is evaluated as $\mathbf{x}^\mathrm{E} \prec \mathbf{x}^\mathrm{D}$. It is clear from this evaluation result that $\mathbf{x}^\mathrm{E}$ is not the best. However, we cannot still say which is the best between $\mathbf{x}^\mathrm{A}$ and $\mathbf{x}^\mathrm{D}$ (since the available information is $\mathbf{x}^\mathrm{C} \prec \mathbf{x}^\mathrm{B} \prec \mathbf{x}^\mathrm{A}$, $\mathbf{x}^\mathrm{C} \prec \mathbf{x}^\mathrm{D}$ and $\mathbf{x}^\mathrm{E} \prec \mathbf{x}^\mathrm{D}$). If $\mathbf{x}^\mathrm{A}$ is evaluated after $\mathbf{x}^\mathrm{E}$, the evaluation result is $\mathbf{x}^\mathrm{A} \prec \mathbf{x}^\mathrm{E}$. From this result, we can say that $\mathbf{x}^\mathrm{D}$ is the best solution. This example explains the necessity of solution re-evaluation to identify the best solution among the examined ones.

In our IEC model, the upper limit on the total number of evaluations is pre-specified (e.g., 200 in our computational experiments). An important requirement in our IEC model is that the best solution among the examined ones should be identified after the pre-specified number of evaluations without any additional re-evaluations. Let us assume that the upper limit on the total number of evaluations is seven in the above-mentioned example. The best solution $\mathbf{x}^\mathrm{D}$ was identified after six evaluations in the order of $\mathbf{x}^\mathrm{A}{} \mathbf{x}^\mathrm{B}{} \mathbf{x}^\mathrm{C}{} \mathbf{x}^\mathrm{D}\mathbf{x}^\mathrm{E}{} \mathbf{x}^\mathrm{A}$. Since the total number of evaluations is six and its upper limit is seven, we can evaluate one more solution $\mathbf{x}^\mathrm{F}$ in comparison with the previously evaluated solution $\mathbf{x}^\mathrm{A}$. If the evaluation result is $\mathbf{x}^\mathrm{F}\prec \mathbf{x}^\mathrm{A}$, we can say that $\mathbf{x}^\mathrm{D}$ is the best solution among the examined six solutions. If the evaluation result is $\mathbf{x}^\mathrm{A}\prec \mathbf{x}^\mathrm{F}$, we cannot say which is better between $\mathbf{x}^\mathrm{D}$ and $\mathbf{x}^\mathrm{F}$. In order to identify the best solution between them, we need to re-evaluate $\mathbf{x}^\mathrm{D}$ after the evaluation of $\mathbf{x}^\mathrm{F}$. However, we cannot perform this re-evaluation since the given upper limit on the total number of evaluations is seven. This means that we cannot identify the best solution among the examined six solutions when the evaluation result is $\mathbf{x}^\mathrm{A}\prec \mathbf{x}^\mathrm{F}$. In order to satisfy both requirements (i.e., the upper limit on the total number of evaluations and the identification of the best solutions among the examined ones), we have to terminate the search after the sixth evaluation in the order of $\mathbf{x}^\mathrm{A}{} \mathbf{x}^\mathrm{B}{} \mathbf{x}^\mathrm{C}{} \mathbf{x}^\mathrm{D}\mathbf{x}^\mathrm{E}{} \mathbf{x}^\mathrm{A}$. This example suggests the necessity of early termination before the total number of evaluations reaches the upper limit.

In our IEC model, we assume that the decision maker can always answer the following question: “Is the current solution $\mathbf{x}_t$ at the *t*-th evaluation better than the previous solution $\mathbf{x}_{t-1}$?” When the decision maker thinks that there is no difference between them, we assume that the decision maker’s answer is “Yes”. In our computational experiments on a minimization problem of an objective function $f(\mathbf{x})$, it is assumed that the decision maker’s answer is “Yes” if and only if $f(\mathbf{x}_{t-1}) \ge f(\mathbf{x}_t)$.

Let us denote the given upper limit on the total number of evaluations by *T*. The task in our IEC model is to search for a good solution using up to *T* evaluations. From the assumption (v) in “Background” section, the best solution among the evaluated ones should be identified when an IEC algorithm is terminated. As we have already explained, the algorithm may be terminated before *T* evaluations due to this requirement. In the next section, we discuss the identification of the best solution among the evaluated ones and the termination of an IEC algorithm.

## Our ($\mu +1$)ES-style IEC algorithm

### Archive maintenance rule

Before explaining our ($\mu +1$)ES-style IEC algorithm, we explain how we can identify the best solution among the examined ones. Let $\mathbf{x}_t$ be the solution to be evaluated at the *t*-th evaluation. We denote a set of candidate solutions for the best solution after the evaluation of $\mathbf{x}_t$ by $S_t$. That is, $S_t$ includes the examined solutions with the possibility to be the best solution. In the following, we first explain the update of $S_t$ depending on the evaluation result of $\mathbf{x}_t$ at the *t*-th evaluation. Then we show how the best solution among the evaluated ones can be identified by re-evaluation.

After the first solution $\mathbf{x}_1$ is evaluated, $S_t$ is specified as $S_1 = \{\mathbf{x}_1\}$ since no other solutions are examined. Next $\mathbf{x}_2$ is examined. If $\mathbf{x}_2$ is better than $\mathbf{x}_1$ (i.e., $\mathbf{x}_1 \prec \mathbf{x}_2$), $S_t$ is updated as $S_2 = \{\mathbf{x}_2\}$ since $\mathbf{x}_2$ is the best solution among the examined one. If $\mathbf{x}_1$ is better than $\mathbf{x}_2$ (i.e., $\mathbf{x}_1 \succ \mathbf{x}_2$), $S_t$ is not changed: $S_2 = S_1 = \{\mathbf{x}_1\}$. Then $\mathbf{x}_3$ is examined. Depending on the evaluation result of $\mathbf{x}_3$, $S_t$ is updated. For example, when $S_2 = \{\mathbf{x}_1\}$ and $\mathbf{x}_2 \prec \mathbf{x}_3$, $S_t$ is updated as $S_3 = \{\mathbf{x}_1, \mathbf{x}_3\}$ since both of $\mathbf{x}_1$ and $\mathbf{x}_3$ have the possibility to be the best solution. In this case, we have two options about the choice of the fourth solution $\mathbf{x}_4$: one is to generate a new solution, and the other is to re-evaluate the first solution $\mathbf{x}_1$ to decrease the size of $S_t$. When $\mathbf{x}_1$ is re-evaluated as the fourth solution (i.e., $\mathbf{x}_4 = \mathbf{x}_1$), $S_t$ is updated as follows: $S_4 = \{\mathbf{x}_4\}$ if $\mathbf{x}_3 \prec \mathbf{x}_4$, and $S_4 = \{\mathbf{x}_3\}$ if $\mathbf{x}_3 \succ \mathbf{x}_4$. When a new solution $\mathbf{x}_4$ is evaluated (instead of re-evaluating $\mathbf{x}_1$) in the case of $S_3 = \{\mathbf{x}_1, \mathbf{x}_3\}$, $S_t$ is updated as follows: $S_4 = \{\mathbf{x}_1, \mathbf{x}_4\}$ if $\mathbf{x}_3 \prec \mathbf{x}_4$, and $S_4 = \{\mathbf{x}_1, \mathbf{x}_3\}$ if $\mathbf{x}_3 \succ \mathbf{x}_4$.

Let us denote the cardinality of $S_t$ by $|S_t|$ (i.e., $|S_t|$ is the number of candidate solutions in $S_t$). The update of $S_t$ based on the evaluation result of $\mathbf{x}_t$ is summarized as follows:

**Case A:**$\mathbf{x}_t$ is a new solution:

1. If $\mathbf{x}_{t-1} \in S_{t-1}$ and $\mathbf{x}_{t-1} \prec \mathbf{x}_t$, then $S_t = S_{t-1} - \{\mathbf{x}_{t-1}\} + \{\mathbf{x}_t\}$. Thus $|S_t| = |S_{t-1}|$.
2. If $\mathbf{x}_{t-1} \in S_{t-1}$ and $\mathbf{x}_{t-1} \succ \mathbf{x}_t$, then $S_t = S_{t-1}$. Thus $|S_t| = |S_{t-1}|$.
3. If $\mathbf{x}_{t-1} \notin S_{t-1}$ and $\mathbf{x}_{t-1} \prec \mathbf{x}_t$, then $S_t = S_{t-1} + \{\mathbf{x}_t\}$. Thus $|S_t| = |S_{t-1}| + 1$.
4. If $\mathbf{x}_{t-1} \notin S_{t-1}$ and $\mathbf{x}_{t-1} \succ \mathbf{x}_t$, then $S_t = S_{t-1}$. Thus $|S_t| = |S_{t-1}|$.

**Case B:**$\mathbf{x}_t$ is a re-evaluation of $\mathbf{x}_q$ ($q < t - 1$ and $\mathbf{x}_t = \mathbf{x}_q$):

1. If $\mathbf{x}_{t-1} \in S_{t-1}$ and $\mathbf{x}_{t-1} \prec \mathbf{x}_t$, then $S_t = S_{t-1} - \{\mathbf{x}_{t-1}, \mathbf{x}_q\} + \{\mathbf{x}_t\}$. Thus $|S_t| = |S_{t-1}| - 1$.
2. If $\mathbf{x}_{t-1} \in S_{t-1}$ and $\mathbf{x}_{t-1} \succ \mathbf{x}_t$, then $S_t = S_{t-1} - \{\mathbf{x}_q\}$. Thus $|S_t| = |S_{t-1}| - 1$.
3. If $\mathbf{x}_{t-1} \notin S_{t-1}$ and $\mathbf{x}_{t-1} \prec \mathbf{x}_t$, then $S_t = S_{t-1} - \{\mathbf{x}_q\} + \{\mathbf{x}_t\}$. Thus $|S_t| = |S_{t-1}|$.
4. If $\mathbf{x}_{t-1} \notin S_{t-1}$ and $\mathbf{x}_{t-1} \succ \mathbf{x}_t$, then $S_t = S_{t-1} - \{\mathbf{x}_q\}$. Thus $|S_t| = |S_{t-1}| - 1$.

Since $\mathbf{x}_t = \mathbf{x}_{q}$ holds in Case B, $S_t$ in B-1 and B-3 can be also written as $S_t = S_{t-1} - \{\mathbf{x}_{t-1}\}$ and $S_t = S_{t-1}$, respectively. The above formulations of $S_t$ in B-1 and B-3 are for explicitly explaining that $\mathbf{x}_t \in S_t$ always holds after the candidate solution set update when $\mathbf{x}_{t-1} \prec \mathbf{x}_t$ (see also A-1 and A-3).

The evaluation of a new solution in Case A increases the number of candidate solutions only in A-3. In Case B, the number of candidate solutions can be decreased by the re-evaluation of a candidate solution whenever $\mathbf{x}_{t-1} \in S_{t-1}$ holds (i.e., in B-1 and B-2). Only in B-3, the re-evaluation of a candidate solution in Case B does not decrease the number of candidate solutions. However, in B-3, $\mathbf{x}_t \in S_t$ always holds after the re-evaluation of $\mathbf{x}_t$. As a result, the re-evaluation at the ($t+1$)th evaluation always decreases the number of candidate solutions. This means that the number of candidate solutions can be always decreased by iterating the re-evaluation twice.

Let us discuss whether a new solution $\mathbf{x}_t$ can be evaluated at the *t*-th evaluation. As explained in “Our IEC model” section, the upper limit on the total number of evaluations is given and denoted by *T*. First, let us consider the case of $\mathbf{x}_{t-1} \in S_{t-1}$. In this case, the evaluation of a new solution $\mathbf{x}_t$ at the *t*-th evaluation does not increase the number of candidate solutions (see A-1 and A-2). After the *t*-th evaluation, the upper limit on the number of remaining evaluations is $(T - t)$. Since one candidate solution can be removed by iterating the re-evaluation twice, we can remove $Int((T - t)/2)$ candidate solutions by iterating the re-evaluation $(T - t)$ times after the *t*-th evaluation where $Int((T - t)/2)$ is the integer part of $(T - t)/2$. Thus we can evaluate a new solution $\mathbf{x}_t$ when the following relation holds: $|S_{t-1}| \le Int((T - t)/2)+1$, i.e., $|S_{t-1}| \le Int((T - t + 2)/2)$. Since the left hand side is also integer, this inequality relation is equivalent to $|S_{t-1}| \le (T - t + 2)/2$.

Next, let us consider the case of $\mathbf{x}_{t-1} \notin S_{t-1}$. In this case, the evaluation of a new solution $\mathbf{x}_{t}$ at the *t*-th evaluation increases the number of candidate solutions from $|S_{t-1}|$ to $|S_t| = |S_{t-1}| + 1$ when the conditions in A-3 hold. In A-3, $\mathbf{x}_{t} \in S_{t}$ always holds after the evaluation of the new solution $\mathbf{x}_{t}$. Thus the number of candidate solutions can be decreased by the re-evaluation at the ($t+1$)th evaluation from $|S_{t}|$ to $|S_{t+1}| = |S_{t}| - 1 = |S_{t-1}|$. After the ($t+1$)th evaluation, the upper limit on the number of remaining evaluations is $(T - t - 1)$. We can remove $Int((T - t - 1)/2)$ candidate solutions by iterating the re-evaluation $(T - t - 1)$ times after the ($t+1$)th evaluation. Thus we can evaluate a new solution $\mathbf{x}_{t}$ when the following relation holds: $|S_{t-1}| \le Int((T - t - 1)/2) + 1$ (i.e., $|S_{t-1}| \le Int((T - t + 1)/2)$). Since the left hand side is also integer, this inequality condition is equivalent to $|S_{t-1}| \le (T - t + 1)/2$.

These discussions are summarized as the following archive maintenance rule:

#### Archive maintenance rule

A new solution $\mathbf{x}_{t}$ is evaluated at the *t*-th evaluation in the following two cases:

- $$\mathbf{x}_{t-1} \in S_{t-1}$$ and $$|S_{t-1}| \le (T - t + 2)/2$$,
- $$\mathbf{x}_{t-1} \notin S_{t-1}$$ and $$|S_{t-1}| \le (T - t + 1)/2$$.

In all the other cases, $\mathbf{x}_{t}$ should be a candidate solution randomly selected from $S_{t-1}$ (excluding $\mathbf{x}_{t-1}$).

Let us discuss the solution evaluation at $t = T$. That is, let us examine whether our archive maintenance rule is valid for the last evaluation at $t = T$. When $\mathbf{x}_{T-1} \in S_{T-1}$, there are two possibilities: $|S_{T-1}| = 1$ and $|S_{T-1}| = 2$. If $|S_{T-1}| = 1$ [i.e., when (a) is satisfied in the archive maintenance rule], a new solution $\mathbf{x}_{T}$ can be evaluated and compared with $\mathbf{x}_{T-1}$. The final solution is the better one between $\mathbf{x}_{T-1}$ and $\mathbf{x}_{T}$. Thus $|S_{T}| = 1$ is satisfied. If $|S_{T-1}| = 2$ (i.e., when (a) is not satisfied), one candidate solution in $S_{T-1}$ is $\mathbf{x}_{T-1}$. The other candidate solution in $S_{T-1}$ is re-evaluated and compared with $\mathbf{x}_{T-1}$ at $t = T$. The final solution is the better one in this comparison. Thus $|S_{T}| = 1$ is satisfied. When $\mathbf{x}_{T-1} \notin S_{T-1}$, $|S_{T-1}| = 1$ always holds from our archive maintenance rule. In this case, (b) is never satisfied since $|S_{T-1}| = 1$ and $t = T$. Thus a new solution is not examined. Since we have only a single candidate in $S_{T-1}$, its re-evaluation is meaningless. Thus no solution is evaluated at $t = T$. As a result, $|S_{T}| = 1$ holds after the termination of the algorithm.

**Fig. 1:**
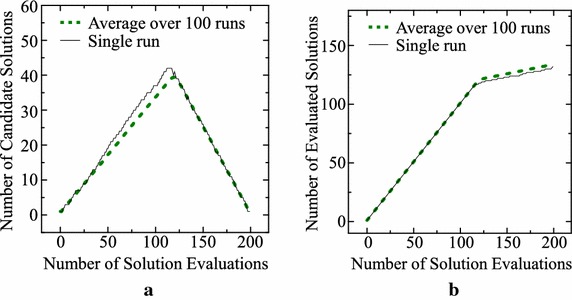
Results of a single run and average results over 100 runs with $T = 200$. **a** The number of candidate solutions. **b** The number of evaluated solutions

For demonstrating our archive maintenance rule, let us perform a simple computer simulation by assuming a minimization problem of $f(x) = x$. We also assume that a new solution $x_t$ is generated as a random real number in the unit interval [0, 1]. Our archive maintenance rule is used for 200 evaluations ($t = 1, 2,\ldots , 200$ and $T = 200$). Average results over 100 runs are shown by dotted lines in Fig. 1. The average number of candidate solutions in $S_t$ and the average number of evaluated new solutions are calculated in Fig. 1a, b, respectively. In Fig. 1, results of a single run are also shown by solid lines. We can see from Fig. 1a that the number of candidate solutions first increases from $|S_t| = 1$ at $t = 1$ to about 40 and then decreases to $|S_T| = 1$ at $T = 200$.

### Archive maintenance for ($\mu +1$)ES-style algorithms

By introducing the upper bound $\mu$ on the number of candidate solutions, we modify our archive maintenance rule in the previous subsection to design a ($\mu +1$)ES-style algorithm. Our idea is to re-evaluate a candidate solution whenever the number of solutions increases from $\mu$ to $(\mu +1)$. That is, a new solution can be evaluated only when the number of candidate solutions is less than or equal to $\mu$. This idea is combined into our archive maintenance rule as follows:

#### Archive maintenance rule for $(\mu +1)$ES-style algorithms

A new solution $\mathbf{x}_{t}$ is evaluated at the *t*-th evaluation in the following two cases:

- $$\mathbf{x}_{t-1} \in S_{t-1}$$ and $$|S_{t-1}| \le \text{ min }\{(T - t + 2)/2, \mu \}$$,
- $$\mathbf{x}_{t-1} \notin S_{t-1}$$ and $$|S_{t-1}| \le \text{ min }\{(T - t + 1)/2, \mu \}$$.

In all the other cases, $\mathbf{x}_{t}$ should be a candidate solution randomly selected from $S_{t-1}$ (excluding $\mathbf{x}_{t-1}$).

**Fig. 2:**
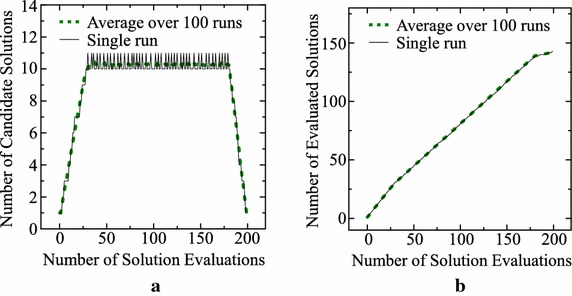
Results of a single run and average results over 100 runs with $\mu = 10$ and $T = 200$. **a** The number of candidate solutions. **b** The number of evaluated solutions

For demonstrating the effect of incorporating the upper bound $\mu$ into our archive maintenance rule, we specify $\mu$ as $\mu =10$ and perform the same computer simulation as in Fig. 1. Average results over 100 runs are shown in Fig. 2 together with results of a single run. As shown in Fig. 2a, the number of candidate solutions is decreased to 10 by re-evaluating a candidate solution whenever it becomes 11. In the final stage, the number of candidate solutions is decreased to one. A little bit more new solutions are examined in Fig. 2b than Fig. 1b. For examining this issue, we perform the same computer simulation for each of the following six settings of $\mu$: $\mu = 1, 2, 5, 10, 20, 50$. The average total number of examined new solutions over 100 runs for each setting is as follows: 146.8, 146.1, 144.6, 142.5, 138.8, 134.2 for $\mu = 1, 2, 5, 10, 20, 50$, respectively. A little bit more new solutions are examined when we use a small value of $\mu$ (i.e., a little bit more re-evaluations are needed when we use a large value of $\mu$).

### Generation of new solutions

An important issue in the design of ($\mu +1$)ES-style algorithms is how to generate a new solution $\mathbf{x}_{t}$ to be compared with the previous solution $\mathbf{x}_{t-1}$ at the *t*-th evaluation. A simple idea is the use of a mutation operator to generate a new solution $\mathbf{x}_{t}$ from a randomly selected candidate solution in $S_{t-1}$. We used this idea in a ($1+1$)ES-style algorithm in Ishibuchi et al. (2012) and a ($\mu +1$)ES-style algorithm in Ishibuchi et al. (2014a). The basic framework of our ($\mu +1$)ES-style algorithm in Ishibuchi et al. (2014a) can be written as follows:

#### The basic framework of our $(\mu +1)$ES-style IEC algorithm

1. An initial solution $\mathbf{x}_{1}$ is randomly generated. Initialize *t* and $S_t$ as $t = 1$ and $S_t = \{\mathbf{x}_{1}\}$.
2. Update *t* as $$t + 1$$ (i.e., $$t = t + 1$$).
3. Decide whether a new solution can be evaluated at the *t*-th evaluation using the archive maintenance rule in “[Sec Sec9]” section.
4. If a new solution can be evaluated, $\mathbf{x}_{t}$ is generated by a mutation operator from a randomly selected candidate solution in $S_{t-1}$. Otherwise, $\mathbf{x}_{t}$ is randomly selected from $S_{t-1} - \{\mathbf{x}_{t-1}\}$.
5. Compare $\mathbf{x}_{t}$ with $\mathbf{x}_{t-1}$. Then update $S_t$ based on the comparison result.
6. If the termination condition is not satisfied, return to Step 2.

When two or more candidate solutions are stored in $S_{t-1}$, it is possible to use a crossover operator as in standard genetic algorithms to generate a new solution $\mathbf{x}_{t}$ in Step 4. That is, a crossover operator is applied to a randomly selected pair of different candidate solutions for generating an offspring. Then a mutation operator is applied to the offspring to generate a new solution $\mathbf{x}_{t}$. It should be noted that we cannot use any fitness-based parent selection mechanism since no information is available about the fitness of each candidate solution (i.e., since no comparison has been performed among the candidate solutions in $S_{t-1}$). Thus, each parent is randomly selected from the candidate solution set. When we use a crossover operator, we always select a pair of different candidate solutions. This is to make the crossover operator always meaningful.

### Computational experiments by our ($\mu +1$)ES-style IEC algorithm

In this subsection, we examine the search ability of our ($\mu +1$)ES-style IEC algorithm under various specifications of $\mu$ on well-known six continuous test problems: Sphere, Rosenbrock, Griewank, Ackley, Levy and Rastrigin functions (e.g., see Surjanovic and Bingham 2013). The number of decision variables is specified as 50: $\mathbf{x} = (x_1, x_2,\ldots , x_n)$ where $n = 50$. This 50-dimensional decision vector is represented by a real number string of length 50 in our computational experiments. The upper limit on the total number of evaluations is always specified as $T = 200$ throughout this paper. Four specifications of $\mu$ are examined: $\mu = 1, 2, 5, 10$.

We examine the search ability of our ($\mu +1$)ES-style IEC algorithm for each combination of the four values of $\mu$ and the two settings for new solution generation mechanisms explained in the previous subsection (i.e., mutation only and crossover & mutation). For mutation, we use the polynomial mutation operator with $P_m = 1$ and $\eta _m = 20$ [for details, see Hamdan (2010)]. For crossover, we use the simulated binary crossover (SBX) with $\eta _c = 15$ (Deb and Kumar 1995). When a new solution is to be generated by mutation only, the polynomial mutation is used with the probability 1.0. When a new solution is to be generated by crossover & mutation, both the SBX crossover and the polynomial mutation are used with the probability 1.0.

The comparison of the current solution $\mathbf{x}_{t}$ with the previous one $\mathbf{x}_{t-1}$ is simulated by a test function $f(\mathbf{x})$ as follows: $\mathbf{x}_{t}$ is preferred to $\mathbf{x}_{t-1}$ by the decision maker when $f(\mathbf{x}_{t}) \le f(\mathbf{x}_{t-1})$ for the minimization problem of $f(\mathbf{x})$. That is, the evaluation result is $\mathbf{x}_{t-1} \prec \mathbf{x}_{t}$ when $f(\mathbf{x}_{t}) \le f(\mathbf{x}_{t-1})$.

Each test problem is a minimization problem of the following non-linear function [16]:

- **Sphere:**$$\begin{aligned} \displaystyle f(\mathbf{x}) = \sum _{i=1}^{n}x_{i}^{2},\quad \hbox { where } -5.12 \le x_i \le 5.12. \end{aligned}$$
- **Rosenbrock:**$$\begin{aligned} \displaystyle f(\mathbf{x}) = \sum _{i=1}^{n-1} [100(x_{i+1} - x_i^2)^2 + (1-x_i)^2],\quad \hbox { where } -2.048 \le x_i \le 2.048. \end{aligned}$$
- **Griewank:**$$\begin{aligned} \displaystyle f(\mathbf{x}) = 1 + \frac{1}{4000} \sum _{i=1}^n x^2_i - \prod _{i=1}^n \text{ cos }\left( \frac{x_i}{\sqrt{i}}\right) ,\quad \hbox { where } -512 \le x_i \le 512. \end{aligned}$$
- **Ackley:**$$\begin{aligned} \displaystyle f(\mathbf{x})= & {} -20 \text{ exp }\left( -0.2 \sqrt{\frac{1}{n} \sum _{i=1}^n x_i^2}\right) - \text{ exp }\left( \frac{1}{n} \sum _{i=1}^n \text{ cos }(2\pi x_i)\right) \\&+ 20 + \text{ exp }(1), \quad \hbox { where } -5 \le x_i \le 5. \end{aligned}$$
- **Levy:**$$\begin{aligned} \displaystyle f(\mathbf{x})= & {} \text{ sin }^2(\pi \omega _1) + \sum _{i=1}^{n-1}(\omega _i - 1)^2\left[ 1+10\text{ sin }^2(\pi \omega _i + 1)\right] \\&+ (\omega _n - 1)^2\left[ 1 + \text{ sin }^2(2 \pi \omega _n)\right] , \\&\quad \hbox { where } \omega _i=1 + (x_i - 1)/4 \hbox { and } -100 \le x_i \le 100. \end{aligned}$$
- **Rastrigin:**$$\begin{aligned} \displaystyle f(\mathbf{x}) = 10n + \sum _{i=1}^n (x_i^2 -10\text{ cos }(2\pi x_i)),\quad \hbox { where } -5.12 \le x_i \le 5.12. \end{aligned}$$

**Fig. 3:**
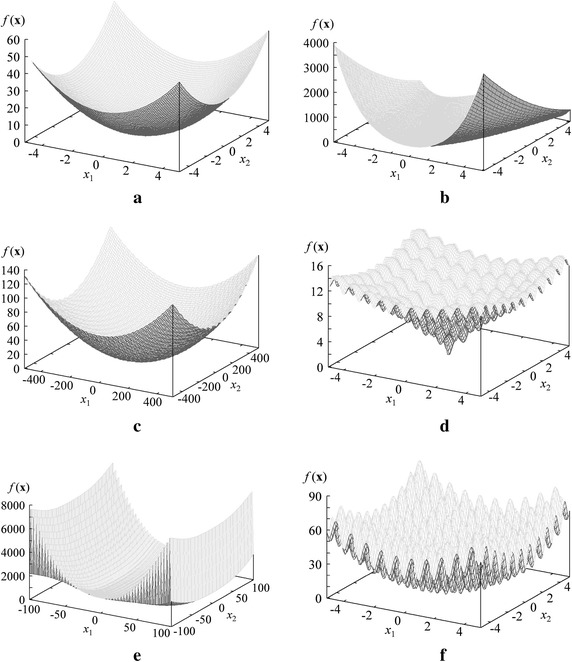
Shape of each test function for the case of two decision variables. **a** Sphere ($-5.12 \le x_i \le 5.12$). **b** Rosenbrock ($-2.048 \le x_i \le 2.048$). **c** Griewank ($-512 \le x_i \le 512$). **d** Ackley ($-5 \le x_i \le 5$). **e** Levy ($-100 \le x_i \le 100$). **f** Rastrigin ($-5.12 \le x_i \le 5.12$)

In Fig. 3, we show the shape of each function for the case of two decision variables [i.e., $\mathbf{x} = (x_1, x_2)$]. The Sphere function is a simple quadratic function with no local minima. The Rosenbrock function has no local minima, either. The decision variables are not separable in the Rosenbrock function whereas they are separable in the Sphere function. The Griewank function has a large number of small local minima. Since they are very small, the function shape in Fig. 3c looks very simple. The Ackley function in Fig. 3d has many small and shallow local minima. The other two functions are complicated non-linear functions with many small but deep local minima as shown in Fig. 3e, f.

From Fig. 3, one may think that near optimal solutions of the Sphere function can be easily found. This is almost always the case in the literature. However, it is not the case in this study due to the following three reasons: (i) the fitness evaluation of each solution is the comparison with the previous solution, (ii) the upper limit on the number of evaluations is only 200, and (iii) each test problem has 50 decision variables. One may also think that multi-point global search algorithms with high diversification ability are needed to handle the highly non-linear Levy and Rastrigin functions. However, for the same three reasons, high convergence ability is very important to find a good solution even for those functions. Our task is to find a good solution of each test problem with 50 decision variables under the severely limited number of evaluations and the very simple fitness evaluation mechanism.

Average results over 1000 runs of our ($\mu +1$)ES-style algorithm are summarized in Tables 1 and 2. Only mutation is used in Table 1 while both crossover and mutation are used in Table 2. No crossover is used when $\mu =1$ even in Table 2. So the same results are shown for $\mu =1$ in the two tables. The best result (i.e., the smallest average function value) for each test problem is highlighted by bold in each table. In these tables, the best or near best results are obtained from our ($\mu +1$)ES-style algorithm with $\mu =1$.

**Table 1:** Average results over 1000 runs under each setting of our ($\mu +1$)ES-style IEC algorithm with only mutation (standard deviations are shown in parentheses)

Problem	$\mu =1$	$\mu =2$	$\mu =5$	$\mu =10$
Sphere	**135.3** (23.7)	169.9 (28.2)	227.9 (31.8)	266.8 (34.9)
Rosenbrock	**4672** (1160)	6066 (1473)	8805 (1946)	11049 (2450)
Griewank	**339.1** (59.3)	425.9 (70.6)	570.8 (79.5)	667.9 (87.2)
Ackley	**7.848** (0.569)	8.102 (0.478)	8.604 (0.412)	8.994 (0.383)
Levy	36818 (5935)	35740 (6094)	**35478** (5566)	36091 (5362)
Rastrigin	734.6 (50.8)	**729.0** (47.6)	729.8 (48.1)	738.7 (46.3)

**Table 2:** Average results over 1000 runs under each setting of our ($\mu +1$)ES-style IEC algorithm with crossover and mutation (standard deviations are shown in parentheses)

Problem	$\mu =1$	$\mu =2$	$\mu =5$	$\mu =10$
Sphere	**135.3** (23.7)	156.0 (26.1)	209.0 (33.1)	250.7 (36.2)
Rosenbrock	**4672** (1160)	5530 (1453)	7925 (1890)	10217 (2320)
Griewank	**339.1** (59.3)	391.1 (65.2)	523.5 (82.7)	627.8 (90.5)
Ackley	**7.848** (0.569)	7.878 (0.495)	8.411 (0.438)	8.841 (0.406)
Levy	36819 (5935)	35407 (6118)	**35033** (5667)	35872 (5312)
Rastrigin	734.6 (50.8)	728.0 (51.1)	**726.6** (44.1)	735.7 (43.3)

**Fig. 4:**
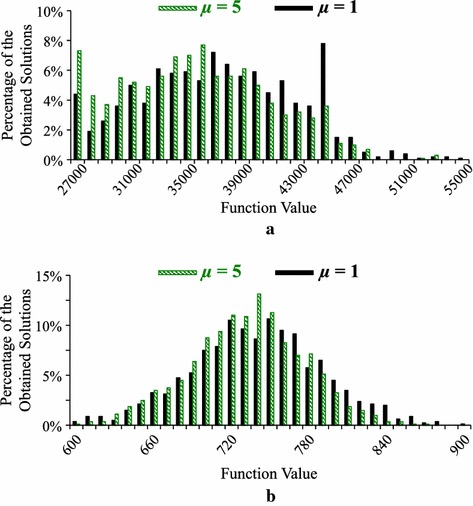
Histogram of 1000 solutions by our (1+1)ES-style algorithm (i.e., $\mu = 1$) with only mutation and our (5+1)ES-style algorithm (i.e., $\mu = 5$) with both crossover and mutation. **a** Levy function. **b** Rastrigin function

For the Levy and Rastrigin functions, the best results are obtained from our ($\mu +1$)ES-style algorithm with $\mu =5$ in Table 2 where both crossover and mutation are used. However, differences between those best results and the results by $\mu =1$ are small in Table 2 if compared with their standard deviations in parentheses. For visually examine their differences, we show the histogram of 1000 solutions obtained from each of the two settings (i.e., $\mu =1$ and $\mu =5$ in Table 2) for the Levy and Rastrigin functions in Fig. 4. We can see that the two histograms by $\mu =1$ and $\mu =5$ for each test problem are heavily overlapping in each plot in Fig. 4. In Fig. 4a, a long black bar around 45,000 may show that the search with $\mu =1$ is trapped in local minima of the Levy function in its many runs.

**Fig. 5:**
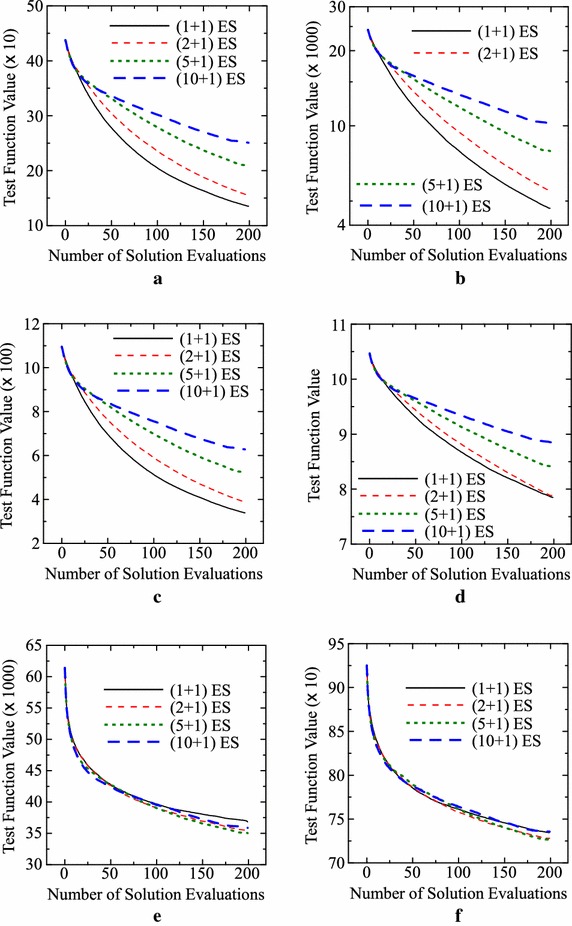
Average results by our algorithm with crossover and mutation for $\mu = 1, 2, 5, 10$. **a** Sphere. **b** Rosenbrock. **c** Griewank. **d** Ackley. **e** Levy. **f** Rastrigin

In Fig. 5, we show how the function value was decreased by 200 evaluations in each setting of our ($\mu +1$)ES-style algorithm with crossover and mutation in Table 2. Figure 5a–d clearly show the deterioration of the search ability by increasing the value of $\mu$ (i.e., by increasing the upper bound on the number of candidate solutions). In Fig. 5e, f, the best results are obtained from $\mu =5$ for the Levy and Rastrigin functions (see Table 2). However, as shown in Fig. 4, we cannot observe any clear performance improvement by increasing the value of $\mu$ in Fig. 5e, f.

## Meta-level approach to the design of IEC algorithms

In our computational experiments in “[Sec Sec6]” section, good results are obtained by the (1+1)ES-style algorithm where new solutions are always generated by mutation. No experimental results strongly support the necessity of multiple candidate solutions and crossover in our ($\mu +1$)ES-style algorithm. In this section, we further try to improve the performance of our ($\mu +1$)ES-style algorithm using an idea of offline meta-level design of IEC algorithms. The necessity of multiple candidate solutions and crossover is clearly shown for the Levy and Rastrigin functions in this section.

In general, an important issue in evolutionary computation is how to generate new solutions to be evaluated. This issue is more important in IEC algorithms since only a small number of solutions can be evaluated. Since re-evaluation of solutions is needed in our IEC model, standard EC algorithms cannot be directly used. Motivated by these discussions, we proposed an idea of offline meta-level design of IEC algorithms in our former study (Ishibuchi et al. 2014b). The basic idea in Ishibuchi et al. (2014b) is to represent an IEC algorithm by an integer string of length *T*. Each string (i.e., each IEC algorithm) is evaluated by applying it to a test problem. In this section, we examine various implementation issues of this idea such as the number of runs for evaluating each string, the string length, and the number of possible operators to generate a new solution.

### Offline meta-level algorithm design approach in Ishibuchi et al. (2014b)

In this subsection, we explain an offline meta-level approach to the design of IEC algorithms in our former study (Ishibuchi et al. 2014b). In our offline meta-level approach, each IEC algorithm with *T* evaluations is coded by a string of length *T* as $\mathbf{\tau } = \tau _1 \tau _2 \ldots \tau _T$ where $\tau _t$ shows how to generate the *t*-th solution $\mathbf{x}_t$. In Ishibuchi et al. (2014b), $\tau _t$ is one of the following six operators:

- Operator 0: Re-evaluation (if inapplicable, random creation is used),
- Operator 1: Re-evaluation (if inapplicable, mutation is used),
- Operator 2: Random creation,
- Operator 3: Crossover (if inapplicable, random creation is used),
- Operator 4: Crossover (if inapplicable, mutation is used),
- Operator 5: Mutation,

where re-evaluation means the random selection of a candidate solution from $S_{t-1}$ (excluding $\mathbf{x}_{t-1}$). If $S_{t-1}$ includes only $\mathbf{x}_{t-1}$ (i.e., $S_{t-1}$ = {$\mathbf{x}_{t-1}$}), re-evaluation is not applicable. In this case, random creation is used in Operator 0 while mutation is used in Operator 1. Mutation is applied to a randomly selected candidate solution from $S_{t-1}$. Except for the generation of the first solution, mutation is always applicable since we have at least one candidate solution. The first solution $\mathbf{x}_{1}$ is always generated by random creation (since all of the other operators are inapplicable to generate the first solution). Crossover is applied to two candidate solutions that are randomly selected from $S_{t-1}$. If the number of candidate solutions in $S_{t-1}$ is one, crossover is not applicable. In this case, random creation is used in Operator 3 while mutation is used in Operator 4.

It should be noted that the string $\mathbf{\tau }$ is used to generate solutions together with our archive maintenance rule in “Archive maintenance rule” section without the upper limit $\mu$ on the number of candidate solutions. More specifically, $\mathbf{\tau }_t$ is used to generate the *t*-th solution $\mathbf{x}_t$ only when the generation of a new solution is allowed by the archive maintenance rule. Otherwise, the re-evaluation of a randomly selected candidate solution from $S_{t-1}$ (excluding $\mathbf{x}_{t-1}$) is performed.

The six operators are denoted by the corresponding integers in Ishibuchi et al. (2014b): $\mathbf{\tau } = \tau _1 \tau _2 \ldots \tau _T$ where $\mathbf{\tau }_t \in \{0, 1, 2, 3, 4, 5\}$ for $t = 1, 2, \ldots , T$. Thus the search space size is $6^T$. A simple evolutionary algorithm with the following components is used to search for the best integer string (i.e., the best IEC algorithm) in Ishibuchi et al. (2014b):

- Random creation of initial strings (i.e., randomly generated initial population),
- Binary tournament selection for choosing a pair of parents,
- Uniform crossover,
- Mutation (the current value is replaced with a randomly specified integer),
- ($\mu +1$)ES-style generation update mechanism to construct the next population.

The fitness of each string is evaluated by applying the corresponding IEC algorithm to a test problem (as in our computational experiments in “[Sec Sec12]” section). In Ishibuchi et al. (2014b), the average result over 100 runs of the IEC algorithm on the test function is used as its fitness value.

### Various implementation issues of offline meta-level approach

In this section, we discuss various implementation issues of our offline meta-level approach to the design of IEC algorithms. The effect of each implementation issue on the performance of designed IEC algorithms is reported in the next subsection.

#### The number of possible operators

In Ishibuchi et al. (2014b), one of the six operators is used to generate a new solution for each evaluation. It is possible to use a different set of operators in our approach. For example, Operator 3 and Operator 4 can be removed for designing an IEC algorithm with re-evaluation, random creation and mutation. It is also possible to add “crossover & mutation” to the set of the six operators in Ishibuchi et al. (2014b). We examine the use of a different set of operators in the next subsection.

#### The number of runs used for evaluating each string

In Ishibuchi et al. (2014b), each string (i.e., each IEC algorithm) is evaluated by the average performance over its 100 runs. In general, the fitness evaluation becomes more accurate by increasing the number of runs. However, the increase in the number of runs leads to the increase in computation time. We examine the effect of the number of runs for the fitness evaluation on the performance of obtained IEC algorithms in the next subsection.

#### The string length

In Ishibuchi et al. (2014b), an IEC algorithm with 200 evaluations is coded by an integer string of length 200. This is to use a different operator to generate a new solution at each evaluation. Since we have six operators, the search space size is $6^{200}$. One may think that we do not have to use a different operator to generate a solution at each evaluation. If we use the same operator for 10 evaluations, the string length is decreased from 200 to 20 as $\mathbf{\tau } = \tau _1 \tau _2 \ldots \tau _{20}$ where $\mathbf{\tau }_t$ is used to generate 10 solutions from the ($10t-9$)-th evaluation to the 10*t*-th evaluation. In the next subsection, we examine various specifications of string length (i.e., various specifications of the number of evaluations where the same operator is used).

### Computational experiments of meta-level algorithm design

In our previous study (Ishibuchi et al. 2014b), our offline meta-level approach was applied to the Sphere and Rastrigin functions under the following setting, which is referred to as the basic setting in this paper:

- Coding: integer string of length 200 with 0, 1, 2, 3, 4, 5,
- Population size: 100,
- Termination condition: 1000 generations,
- Generation update model: ($\mu +1$)ES-style,
- Crossover: uniform crossover with the crossover probability 1.0,
- Mutation: random generation of an integer value with the mutation probability 1/(string length),
- Fitness evaluation of each string: average performance of 100 runs.

In this paper, we apply our approach to all the six test problems in “[Sec Sec6]” section. Average results are calculated over ten runs of our approach. After the termination of our approach, a single string with the best fitness value in the final population is selected as the designed IEC algorithm. The designed IEC algorithm is evaluated by its additional 100 runs which are different from the 100 runs for fitness evaluation during the execution of our offline meta-level approach. The design of an IEC algorithm and its performance evaluation are iterated ten times. This means that the performance of our approach is evaluated by 1000 runs (i.e., 100 runs of each of the ten algorithms designed by our approach).

First, let us examine the effect of a set of operators for solution generation on the performance of designed algorithms. As explained in “Offline meta-level algorithm design approach in Ishibuchi et al. (2014b)” section, the six operators are used to generate new solutions in our former study (Ishibuchi et al. 2014b). In this paper, we also examine the following two settings with respect to possible operators in addition to the six operators in Ishibuchi et al. (2014b).

#### Four operators

In order to examine the necessity of crossover, we perform computational experiments using the set of the following four operators.

- Operator 0: Re-evaluation (if inapplicable, random creation is used),
- Operator 1: Re-evaluation (if inapplicable, mutation is used),
- Operator 2: Random creation,
- Operator 5: Mutation.

#### Eight operators

For comparison, we also perform computational experiments using the following two operators in addition to the six operators in “Offline meta-level algorithm design approach in Ishibuchi et al. (2014b)” section (eight operators in total).

- Operator 6: Crossover & Mutation (if crossover is inapplicable, random creation is used),
- Operator 7: Crossover & Mutation (if crossover is inapplicable, mutation is used).

Average results over ten runs are summarized in Table 3. For comparison, we show the average results by the (1+1)ES-style algorithm in the second column of Table 3. The best average result for each test problem is highlighted by bold. We cannot observe any clear performance improvement from the (1+1)ES-style algorithm for the first four test problem in Table 3. This observation is consistent with the performance deterioration for those test problems by increasing the value of $\mu$ in “[Sec Sec6]” section. For the last two test problems, however, we can observe clear performance improvement by our approach.

**Fig. 6:**
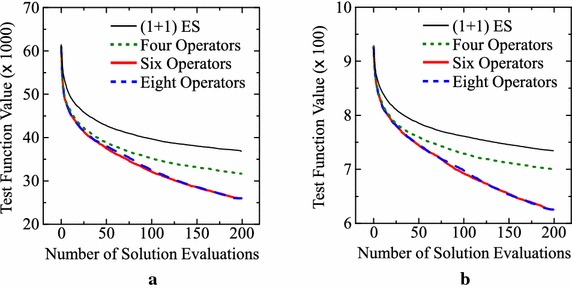
Average results of the designed IEC algorithms for the three settings of the operators. **a** Levy function. **b** Rastrigin function

As we have already explained, our approach is applied to each test problem 10 times. Each of the ten designed algorithms is evaluated by its 100 runs after the termination of our approach (i.e., 1000 runs in total for each test problem). Figure 6 shows average results over those 1000 runs for the Levy and Rastrigin functions. For comparison, we also show the average results over 1000 runs of the (1+1)ES-style algorithm in “[Sec Sec6]” section. In Fig. 6, we can observe clear performance improvement by our approach with the six and eight operators. Inferior performance of the four-operator setting in comparison with the six-operator and eight-operator settings in Fig. 6 suggests the usefulness of crossover for the Levy and Rastrigin functions.

**Table 3:** Average results over 10 runs of our offline meta-level approach with a different setting of solution generation operators

Test problem	(1+1)ES-style IEC with $\mu =1$	Meta-level algorithm design approach		
		Four operators	Six operators	Eight operators
Sphere	**135.3** (23.7)	138.2 (24.3)	140.4 (26.0)	143.6 (25.1)
Rosenbrock	**4672** (1160)	4753 (1204)	4742 (1257)	4866 (1246)
Griewank	**339.1** (59.3)	346.5 (60.8)	349.8 (61.6)	361.4 (64.9)
Ackley	**7.848** (0.569)	7.863 (0.523)	7.885 (0.516)	7.878 (0.500)
Levy	36819 (5935)	31682 (4697)	26023 (4255)	**25946** (4274)
Rastrigin	734.6 (50.8)	700.5 (38.4)	625.8 (43.7)	**625.5** (43.3)

Standard deviations in parentheses are calculated over 1000 runs by the ten designed IEC algorithms for each problem

**Fig. 7:**
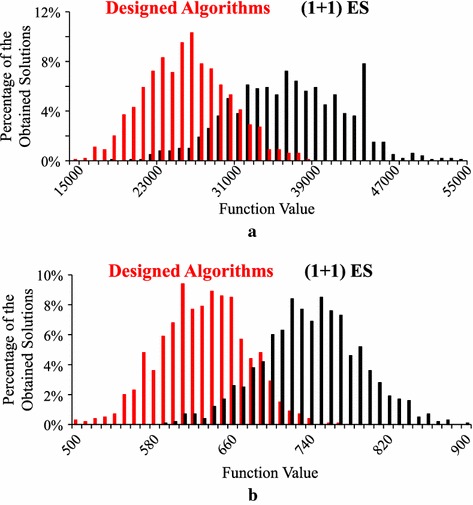
Histogram of 1000 solutions by the ten designed algorithms with the six-operator setting and the (1+1)ES-style algorithm in “[Sec Sec6]” section. **a** Levy function. **b** Rastrigin function

In Fig. 7, we show the histogram of 1000 solutions obtained by 100 runs of each of the ten designed algorithms with the six-operator setting. For comparison, we also show the histogram of 1000 solutions by the ($1+1$)ES-style algorithm in “[Sec Sec6]” section. We can observe clear differences between the two histograms in each plot in Fig. 7.

**Fig. 8:**
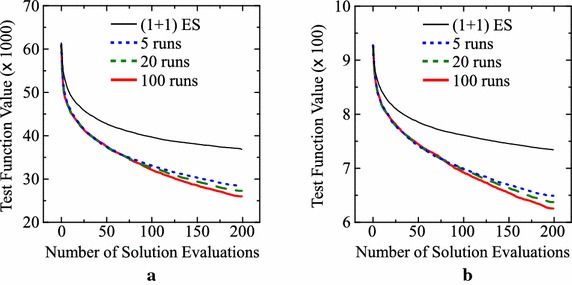
Average results of over 1000 runs of the ten designed IEC algorithms (100 runs of each algorithm) for the three settings of the number of runs for fitness evaluation. **a** Levy function. **b** Rastrigin function

Next, let us examine the effect of the number of runs for fitness evaluation on the performance of our offline meta-level approach. In the previous computational experiments, each string (i.e., each IEC algorithm) is evaluated by its 100 runs on a test problem. That is, the average result over the 100 runs is used as the fitness of each string. It is likely that the decrease in the number of runs for fitness evaluation leads to the performance deterioration of designed IEC algorithms. For discussing this issue, we perform computational experiments for three settings: 5 runs, 20 runs and 100 runs for fitness evaluation. All the other specifications are the same as the basic setting (e.g., the six operators for solution generation). Our approach is applied to each test problem ten times using each setting of the number of runs for fitness evaluation. Average experimental results are summarized in Table 4. Experimental results on the Levy and Rastrigin functions are also shown in Fig. 8. As expected, the performance of the designed IEC algorithms was deteriorated by decreasing the number of runs. However, the deterioration is not so severe if compared with the improvement from the (1+1)ES-style algorithm for the Levy and Rastrigin functions as shown in the last two rows of Table 4 and Fig. 8.

**Table 4:** Average results over 10 runs of our offline meta-level approach with a different setting of the number of runs for fitness evaluation

Test problem	(1+1)ES-style IEC with $\mu =1$	Meta-level algorithm design approach		
		5 runs	20 runs	100 runs
Sphere	**135.3** (23.7)	158.1 (27.8)	147.3 (25.7)	140.4 (26.0)
Rosenbrock	**4672** (1160)	5623 (1344)	5046 (1281)	4742 (1257)
Griewank	**339.1** (59.3)	398.7 (68.9)	372.3 (64.7)	349.8 (61.6)
Ackley	**7.848** (0.569)	8.203 (0.462)	7.968 (0.498)	7.885 (0.516)
Levy	36819 (5935)	28403 (4504)	27281 (4419)	**26023** (4255)
Rastrigin	734.6 (50.8)	649.2 (42.4)	637.5 (41.1)	**625.8** (43.7)

Standard deviations in parentheses are calculated over 1000 runs of the ten designed IEC algorithms (100 runs of each algorithm) for each problem

Finally, let us examine the effect of string length on the performance of our meta-level algorithm design approach. In our previous computational experiments, an IEC algorithm with 200 evaluations is coded by an integer string $\mathbf{\tau }$ of length 200 as $\mathbf{\tau } = \tau _1 \tau _2 \ldots \tau _{200}$ where $\mathbf{\tau }_t$ is used to generate a solution for the *t*-th evaluation. When we use the six operators, the total number of strings is $6^{200}$. One may think that the problem size (i.e., $6^{200}$) may be too large. One may also think that it is not needed to use a different operator for generating each solution. The string length can be decreased by using $\mathbf{\tau }_t$ for generating multiple solutions. In this paper, we examine the following four settings: $\mathbf{\tau }_t$ is used for generating a single solution (i.e., the basic setting: string length 200), 5 solutions (string length 40), 10 solutions (string length 20), and 50 solutions (string length 4). Each setting is evaluated by ten runs of our offline meta-level approach.

**Fig. 9:**
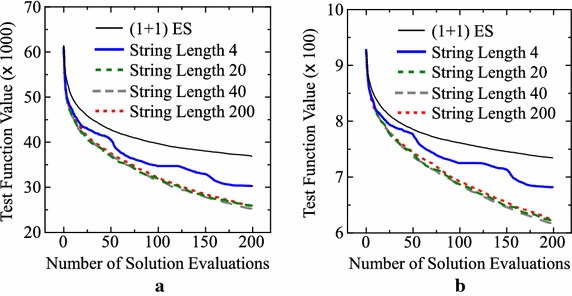
Average results of the designed IEC algorithms for the four settings of the string length. **a** Levy function. **b** Rastrigin function

Experimental results are summarized in Table 5. For the first four test problems, similar results are obtained from the four settings of the string length and the (1+1)ES-style IEC algorithm in Table 5. This observation may suggest that we do not have to use different operators for those test problems (i.e., only mutation is enough). This issue will be further discussed later in “Algorithm design” section. For the Levy and Rastrigin functions, however, we can observe clear performance deterioration when the string length is specified as 4. Experimental results on the Levy and Rastrigin functions are also shown in Fig. 9. In the case of string length 4, the same operator continues to be used to generate 50 solutions. That is, solution generation operators are changed only after the 50th, 100th and 150th evaluations. This leads to an interesting shape of the solid blue line in each plot in Fig. 9. For example, we can observe slow performance improvement before the 50th evaluation and speed-up after the 50th evaluation in Fig. 9a, b. Since almost the same results are obtained from the other settings (i.e., string length of 20, 40, 200), we can see that a different operator is needed for every ten solutions (whereas a different operator is not needed for every solution).

**Table 5:** Average results over 10 runs of our offline meta-level approach with a different setting of the string length

Test problem	(1+1)ES-style IEC with $\mu =1$	Meta-level algorithm design approach			
		Length 4	Length 20	Length 40	Length 200
Sphere	135.3 (23.7)	136.8 (22.5)	**129.1** (21.8)	130.0 (22.4)	140.4 (26.0)
Rosenbrock	4672 (1160)	4490 (1164)	**4447** (1092)	4490 (1152)	4742 (1257)
Griewank	339.1 (59.3)	342.9 (56.3)	**323.7** (54.5)	326.8 (56.9)	349.8 (61.6)
Ackley	7.848 (0.569)	7.818 (0.537)	7.769 (0.520)	**7.761** (0.532)	7.885 (0.516)
Levy	36819 (5935)	30317 (4386)	25928 (4270)	**25326** (4414)	26023 (4255)
Rastrigin	734.6 (50.8)	682.0 (44.5)	622.8 (44.0)	**617.8** (46.3)	625.8 (43.7)

Standard deviations in parentheses are calculated over 1000 runs by the ten designed IEC algorithms for each problem

### Further examination of designed algorithms

As shown in our computational experiments in this section, our offline meta-level approach found better algorithms than the (1+1)ES-Style algorithm for the Levy and Rastrigin functions. In this subsection, we further examine the ten designed algorithms for each test problem by the best setting for each test problem in Table 5 (i.e., six operators, 100 runs and string length 20 or 40).

Each of the ten designed algorithms is an integer string of length 20 for the first three problems (Sphere, Rosenbrock and Griewank) and length 40 for the last three problems (Ackley, Levy and Rastrigin). In Table 6, we show the average percentage of each integer among the generated ten algorithms for each problem.

**Table 6:** Average percentage of each integer among the ten IEC algorithms designed by ten runs of our offline meta-level approach

Problem	0: re-evaluation (random) (%)	1: re-evaluation (mutation) (%)	2: random (%)	3: crossover (random) (%)	4: crossover (mutation) (%)	5: mutation (%)
Sphere	5.0	94.5	0.0	0.0	0.5	0.0
Rosenbrock	4.5	92.5	0.5	0.0	2.5	0.0
Griewank	5.0	94.5	0.0	0.0	0.5	0.0
Ackley	4.0	81.75	1.25	3.75	7.5	1.75
Levy	13.0	22.75	8.0	24.5	28.75	3.0
Rastrigin	17.25	20.75	5.5	30.25	24.25	2.0

Each of the ten designed algorithms for each test problem is applied to the test problem 100 times. During this computational experiment, we monitor how each solution is generated. That is, we check which operator is actually used for generating each solution. Then we calculate the percentage of solutions generated by each operator. Our experimental results are summarized in Table 7. In Table 7, “Re-evaluation (operator)” and “Re-evaluation (archive)” mean the re-evaluation by the designed algorithm string and the archive maintenance rule, respectively.

**Table 7:** Average percentage of each operator over 100 runs of each IEC algorithm designed by ten runs of our offline meta-level approach (i.e., over 1000 runs in total for each test problem)

Problem	Re-evaluation (operator) (%)	Re-evaluation (archive) (%)	Random (%)	Crossover (%)	Mutation (%)
Sphere	28.1	0.1	4.0	0.2	67.6
Rosenbrock	27.2	0.7	4.1	1.2	66.8
Griewank	28.1	0.1	4.0	0.2	67.6
Ackley	27.3	0.7	4.8	7.7	59.5
Levy	28.2	2.3	13.8	45.6	10.1
Rastrigin	27.2	2.1	16.0	45.5	9.2

**Table 8:** Average percentage of each operator in a different phase for the Levy function in Table 7

Search phase	Re-evaluation (operator) (%)	Re-evaluation (archive) (%)	Random (%)	Crossover (%)	Mutation (%)
1–50th evaluations	20.7	0.0	40.8	35.7	2.7
51–100th evaluations	37.2	0.0	4.7	49.4	8.7
101–150th evaluations	28.8	0.0	6.2	49.7	15.4
151–200th evaluations	26.1	9.4	3.3	47.4	13.8
1–200th evaluations	28.2	2.3	13.8	45.6	10.1

**Table 9:** Average percentage of each operator in a different phase for the Rastrigin function in Table 7

Search phase	Re-evaluation (operator) (%)	Re-evaluation (archive) (%)	Random (%)	Crossover (%)	Mutation (%)
1–50th evaluations	21.5	0.0	37.2	37.2	4.1
51–100th evaluations	36.4	0.0	8.3	46.0	9.3
101–150th evaluations	26.5	0.0	11.9	48.7	13.0
151–200th evaluations	24.3	8.3	6.6	50.3	10.5
1–200th evaluations	27.2	2.1	16.0	45.5	9.2

We can observe clear differences in experimental results in Table 7 between the first four problems and the last two problems. Crossover is mainly used to generate new solutions for the last two problems whereas mutation is mainly used for the first four problems. More solutions are generated randomly for the last two problems.

These differences are related to the shape of each function: the Levy and Rastrigin functions have a number of deep local minima. We can also see that the percentage of re-evaluations is almost the same for all test problems. This is because a single best solution should be identified within 200 evaluations. A little bit more re-evaluations are performed by the archive maintenance rule for the Levy and Rastrigin functions. This may be related to the number of candidate solutions (as we mentioned in “[Sec Sec9]” section with respect to the relation between the number of re-evaluations and the upper limit $\mu$ on the number of candidate solutions).

**Fig. 10:**
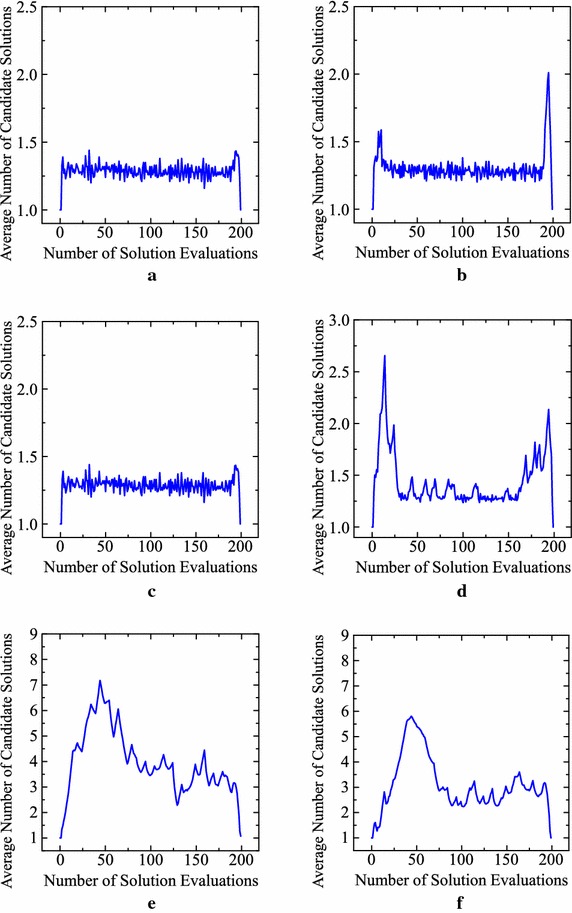
Average number of candidate solutions over 1000 runs of the ten designed algorithms. **a** Sphere. **b** Rosenbrock. **c** Griewank. **d** Ackley. **e** Levy. **f** Rastrigin

For discussing this issue, we calculate the average number of candidate solutions in our computational experiments by the ten designed algorithms for each test problem. Experimental results are shown in Fig. 10. It should be noted that different scales are used for the vertical axis between Fig. 10a–d and e–f. The number of candidate solutions in Fig. 10e and f is much larger than the results for the first four test problems in Fig. 10a–d. This difference may be related to a difference in the average percentage of re-evaluations by the archive maintenance rule in Table 7 between the first four problems and the last two problems.

For the Levy and Rastrigin functions, we further check which operator is actually used to generate each solution. Then we calculate the percentage of each operator in each of the following four different search phases: 1–50th evaluations, 51–100th evaluations, 101–150th evaluations and 151–200th evaluations. Our experimental results are summarized in Tables 8 and  9. We can obtain the following observations from both tables:

1. New solutions for the first 50 evaluations are mainly generated randomly whereas percentages of random creation are very low for the other evaluations (i.e., 51–200th evaluations). This observation suggests that the designed IEC algorithms first search for promising search areas randomly before generating new solutions from stored candidate solutions by crossover.
2. Percentages of re-evaluation in the first 50 evaluations are clearly lower than those in the other evaluations. This observation corresponds to the increase in the number of candidate solutions in Fig. 10e, f during the first 50 evaluations.
3. There exist no large differences in the average percentage of each operator among the last three search phases: 51–100, 101–150 and 151–200 evaluations. That is, the average percentages of mutation, crossover, random generation and re-evaluation (operator) are in [8, 16], [46, 51], [3, 12] and [24, 38], respectively. This observation may suggest the necessity of totally different search strategies between the early exploration phase and the other exploitation phases for the Levy and Rastrigin functions. For comparison, we show experimental results for the Sphere function in Table 10. An interesting observation in Table 10 is a relatively larger percentage of random creation in the first 50 evaluations (i.e., 15.8 %). It seems that the designed algorithms search for good starting points by randomly generating solutions in the early search phase. However, even in the first 50 evaluations, mutation is mainly used in Table 10 for the Sphere function with no local minima.

**Table 10:** Average percentage of each operator in a different phase for the Sphere function in Table 7

Search phase	Re-evaluation (operator) (%)	Re-evaluation (archive) (%)	Random (%)	Crossover (%)	Mutation (%)
1–50th evaluations	28.3	0.0	15.8	0.0	55.9
51–100th evaluations	28.8	0.0	0.0	0.0	71.2
101–150th evaluations	28.0	0.0	0.0	0.0	72.0
151–200th evaluations	27.2	0.6	0.0	1.0	71.2
1–200th evaluations	28.1	0.1	4.0	0.2	67.6

### Algorithm design

From our experimental results, we can see that the first four problems (Sphere, Rosenbrock, Griewank and Ackley) and the last two problems (Levy and Rastrigin) need totally different algorithms. For the first four problems, the ($1+1$)ES-style algorithm worked well. However, from Tables 8, 9, 10, the examination of randomly generated solutions in the early generations seems to be a good idea for not only the last two test problems but also the first four test problems. So, we implement a slightly modified ($1+1$)ES-style algorithm by using random solutions in the first ten evaluations instead of mutated solutions in the ($1+1$)ES-style algorithm. This algorithm is referred to as the “($1+1$)ES-Random-10” algorithm.

**Fig. 11:**
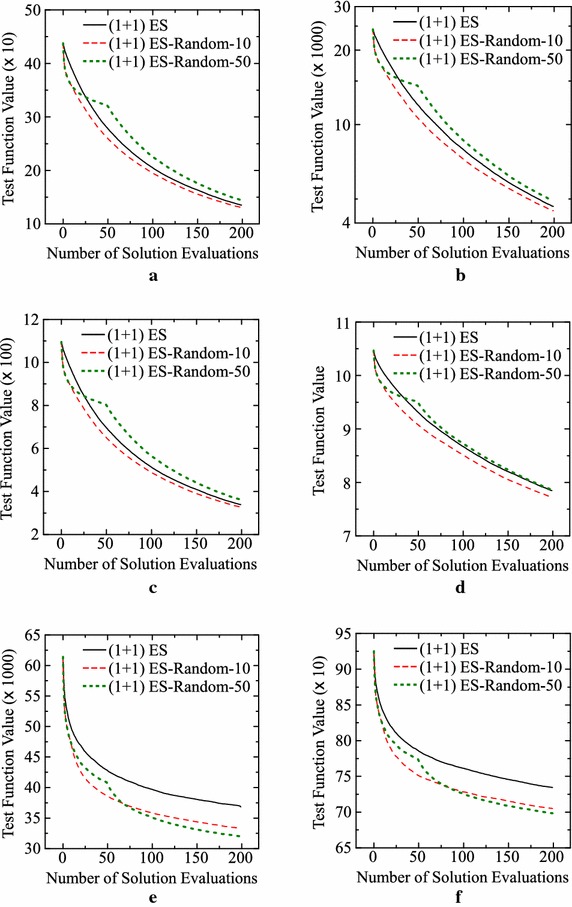
Experimental results by the ($1+1$)ES-style algorithm and its two variants: ($1+1$)ES-Random-10 and ($1+1$)ES-Random-50. **a** Sphere. **b** Rosenbrock. **c** Griewank. **d** Ackley. **e** Levy. **f** Rastrigin

For comparison, we also implement the “($1+1$)ES-Random-50” algorithm where the first 50 solutions are generated randomly. Experimental results are summarized in Fig. 11. It is shown by Fig. 11 that the use of random solutions in the first ten evaluations clearly improves the performance of the ($1+1$)ES-style algorithm for the last two test problems without degrading its performance for the first four test problems. For the last two test problems, we can further improve the performance of the ($1+1$)ES-style algorithm by increasing the archive size and using the crossover operator. However, its performance for the first four test problems is deteriorated by those changes.

**Fig. 12:**
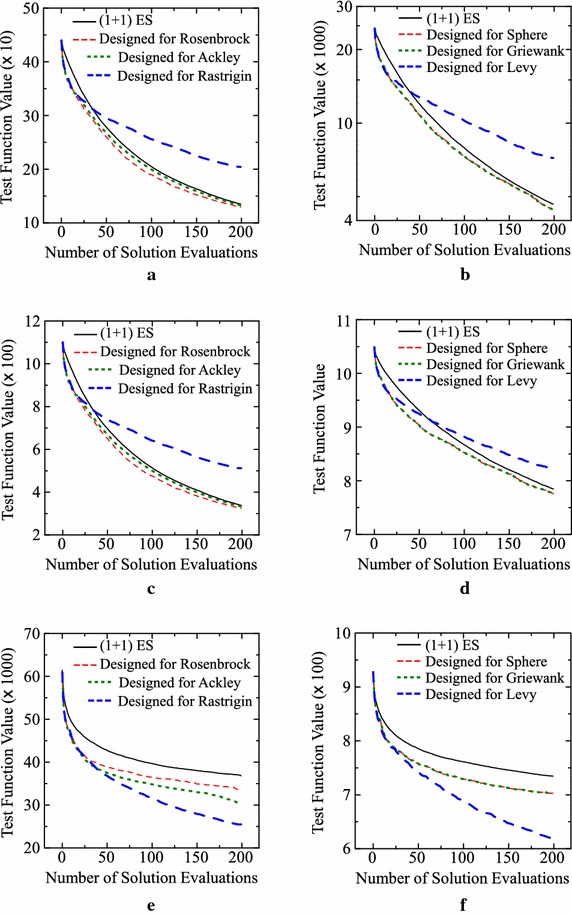
Examination of the generalization ability of the designed algorithms under the best setting in Table 5. Designed algorithms for a test problem in the *left (right) column* is applied to the other test problems in the *right (left) column*. **a** Sphere. **b** Rosenbrock. **c** Griewank. **d** Ackley. **e** Levy. **f** Rastrigin

Finally, we examine the generalization ability of the ten designed algorithms in the best setting in Table 5. Each algorithm designed for a test problem is applied to other test problems for examining its generalization ability. In our computational experiments, we divide our six test problems into two groups: Group A = {Sphere, Griewank, Levy} and Group B = {Rosenbrock, Ackley, Rastrigin}. Group A and Group B include the three test problems in the left and right columns of each figure (e.g., Fig. 11), respectively. Each of the ten algorithms designed for a test problem in one group is applied to each test problem in the other group 100 times. Experimental results are summarized in Fig. 12. We can observe from Fig. 12 that the designed algorithms for one of the last two test problems work well on the other test problem in Fig. 12e, f. That is, the designed algorithms for Levy (Rastrigin) work well on Rastrigin (Levy). However, those algorithms do not work well on the first four test problems in Fig. 12a–d. We can also see that the designed algorithms for one of the first four test problems work well on the other three test problems in Fig. 12a–d. Our experimental results show that the designed algorithms have a limited but high generalization ability to similar test problems.

## Conclusion

We examined the performance of our offline meta-level approach to the design of IEC algorithms. The main feature of our approach is that a different operator is used to generate each solution. In the basic setting of our approach, an IEC algorithm is coded as a string of operators where the string length is the same as the number of solutions to be generated. We obtained promising results where efficient multi-point search algorithms were designed for non-linear test problems with many local minima. The designed algorithms seemed to adjust the diversity-convergence balance over 200 evaluations by frequently changing operators to generate new solutions. With respect to the frequency of operator change, we obtained similar results from the following three settings: the same solution generation operator was used to generate a single, five and ten solutions (Table 5). This observation suggests that we do not need to change operators to generate each solution. However, when we used the same operator to generate 50 solutions, we observed clear performance deterioration of designed algorithms. This observation suggests the need of a more frequent change of operators than every 50 solutions.

As expected, different algorithms were designed for different test problems. One common feature among all the designed algorithms was the use of randomly generated solutions in an early stage of evolution. We demonstrated that the performance of the ($1+1$)ES-style algorithm was improved by using randomly generated solutions in its first ten generations (Fig. 11). We also demonstrated that a designed algorithm for one test problem worked well on another test problem when they were similar to each other with respect to the shape of the fitness function (Fig. 12). This result suggests the possibility of designing a high-performance IEC algorithm for a real-world application problem if we have a similar test problem.

Since this study is just a start of research on offline meta-level algorithm design where a search algorithm is handled as a string of solution generation operators, there exist a large number of future research topics. For example, the usefulness of our IEC model should be evaluated by its applications to real-world IEC problems. Its combination with a brain computer interface is an interesting future research topic. Since the proposed idea of offline meta-level algorithm design is a general framework, it can be applicable to not only continuous test problems but also other problems such as combinatorial and multi-objective problems. The design of an IEC algorithm using a surrogate model seems to be a promising research topic where a surrogate model can be used instead of a test problem for fitness evaluation of IEC algorithms in our meta-level approach.
